# Systemic immune-inflammation index as a prognostic marker in HER2-positive breast cancer patients undergoing trastuzumab therapy

**DOI:** 10.1038/s41598-024-57343-0

**Published:** 2024-03-19

**Authors:** Jian Pang, Nianhua Ding, Nana Yin, Zhi Xiao

**Affiliations:** 1 Department of General Surgery, The Second Xiangya Hospital, Central South University, Changsha, China; 2 Department of Breast Surgery, Xiangya Hospital, Central South University, 87# Xiangya Road, Changsha, 410008 Hunan China; 3 Clinical Research Center for Breast Cancer in Hunan Province, Changsha, China; 4 Department of Clinical Laboratory, The Affiliated Changsha Hospital of Xiangya School of Medicine, Central South University, Changsha, China; 5 Department of Operating Room, First People’s Hospital of Changde, Changde, China

**Keywords:** Breast cancer, HER-2-positive, Trastuzumab, Systemic immune-inflammatory index (SII), Prognosis, Cancer, Breast cancer

## Abstract

The prognostic value of SII (Systemic Immune-Inflammation Index) in HER-2-positive breast cancer (BC) patients, regardless of whether they receive trastuzumab treatment, and its potential value to distinguish patients who may benefit from trastuzumab therapy, warrant further investigation. Clinical data was collected from 797 HER-2-positive BC patients between July 2013 and March 2018. Baseline data differences were adjusted with propensity score matching. Univariate and multivariate analyses explored the correlation between clinical pathological factors, SII, and DFS. Four groups were established. Based on the baseline SII values of the participants, patients who did not receive trastuzumab treatment were divided into Group 1 (Low-SII) and Group 2 (High-SII), where SII had no predictive value for prognosis between groups. Participants who received trastuzumab treatment were also divided into two groups: the Low-SII group (Group 3) and the High-SII group (Group 4). The 5-year DFS was significantly higher in Group 3 than in Group 4 (91.76% vs. 82.76%, P = 0.017). Furthermore, multivariate analysis demonstrated a significant association between high SII and shorter DFS (HR = 3.430, 95% CI = 1.830–6.420, P < 0.001). In HER-2-positive BC patients treated with trastuzumab, those with lower SII showed a longer DFS, suggesting that SII may help in identifying patients who benefit from trastuzumab therapy.

## Introduction

Elevated levels of human epidermal growth factor receptor-2 (HER-2) are seen in between 20 and 25% of breast cancer (BC) cases, resulting in an invasive tumor phenotype and associated with inferior clinical prognosis^1–3^. Trastuzumab (trade name, Herceptin) is a monoclonal antibody targeting the HER-2 extracellular domain and is considered one of the most successful targeted therapies for HER-2 overexpressing BC^4^. It blocks activation of HER-2 signaling and leads to antibody-dependent cell-mediated cytotoxicity (ADCC), thereby reducing recurrence risk by 25% in adjuvant therapy^5,6^. Combining trastuzumab with chemotherapy has significantly improved the prognosis of early and advanced HER-2-positive BC patients^7^. Resistance to Trastuzumab is also common, but its specific mechanism is not fully understood. Therefore, exploring clinical biomarkers to predict sensitivity to trastuzumab has significant clinical value.

With a deeper understanding of BC, the roles of inflammation and immunity in disease progression, drug sensitivity, and prognosis are becoming better recognized^8,9^. Notably, basic immune monitoring and systemic immune status changes can influence both the response to treatment and prognosis in BC patients^10,11^. Moreover, host anti-tumor immunity determines the patient's prognostic outcome^12^. Furthermore, the systemic immune inflammation index (SII), a blood marker that reflects the body’s immune status^13^, has also been widely used in predicting disease-free survival (DFS) in HER-2-positive BC^14–18^.

However, there have been few studies that have evaluated the relationship between the SII and DFS in patients with HER-2-positive BC who are either receiving trastuzumab treatment or no treatment. Therefore, in this retrospective analysis of 797 patients, we aimed to identify prognostic factors and predictive indicators of potential benefits from trastuzumab therapy.

## Patients and methods

### Patient background

This research encompassed 797 women diagnosed with HER-2-positive invasive BC who received treatment in the Department of Breast Surgery, Xiangya Hospital, Central South University, between July 2013 and March 2018. Of these patients, 544 underwent a 1-year trastuzumab treatment, with a loading dose of trastuzumab 8 mg/kg and a maintenance dose of 6 mg/kg every 3 weeks. In contrast, trastuzumab treatment was not administered to the remaining 253 patients. The reason for the lack of trastuzumab treatment was that trastuzumab (Herceptin^®^) was not covered by insurance until July 2016, and these patients could not afford it.

Patient health records were evaluated to collect data including age, medical records, laboratory examinations, and pathological findings, including tumor dimensions, histological grading, lymph node condition, hormone receptor (HR) status, and the postoperative supplementary treatment plan incorporating chemotherapy, radiation, and endocrine therapy.

All patients underwent surgical treatment. This involved modified radical mastectomy, or breast-conserving surgery, together with a biopsy of sentinel lymph nodes. Patients also received endocrine treatment and radiotherapy based on postoperative pathological results following guideline recommendations.

Individuals diagnosed with inflammatory BC, multiple tumors, injuries (acute or chronic), inflammation (acute or chronic), cirrhosis of the liver, hematological disorders, or end-stage kidney disease were not included in the study. The Institutional Review Board of the Xiangya Hospital sanctioned the study (Approval No: 202002021). Moreover, all patients provided informed consent when the database was constructed. Furthermore, all methods were conducted in accordance with the appropriate guidelines and regulations stipulated in the Declaration of Helsinki.

### Pathological characteristics

HR-positivity refers to positivity to either the estrogen (ER) or progesterone (PR) receptors. These are also evaluated by positive staining of the nuclei in a minimum of 1% of invasive tumor cells. Thus, HER-2 positivity is defined based on immunohistochemical staining results of 3+ or 2+ with a HER-2/CEP17 ratio of over 2.2, as measured by fluorescence in situ hybridization.

### Laboratory data

SII was calculated based on the complete blood count results obtained at the patient's initial hospital admission. As such, SII is calculated as the product of the platelet and neutrophil counts divided by the lymphocyte count. Blood samples were collected without clinical signs of fever or infection. In the group not receiving trastuzumab, patients were assigned to low-SII (SII < 446, 126 cases) and high-SII (SII ≥ 446, 127 cases) groups using the median SII value (446) as the threshold. In the trastuzumab treatment group, patients were allocated to low-SII (SII < 430, 272 cases) and high-SII (SII ≥ 430, 272 cases) groups using the median SII value of 430 as the threshold.

### Patient follow-up

Follow-up assessments were conducted after 3–6 months for 2 years following surgery and 6 months for the subsequent three to 10 years. Additionally, patients were followed up by telephone interviews and reviewing outpatient examination data to assess patient prognosis and survival status. Notably, DFS was defined as the time between surgery and the first disease recurrence or death.

### Statistical analysis

Chi-square tests were used for comparison of categorical data, and Kaplan–Meier (KM) curves and log-rank tests were used for comparison of DFS rates. Significant variables in the univariate analysis were incorporated into the multivariate Cox analysis to identify factors impacting DFS.

To minimize selection bias and potential confounding factors between the groups, propensity score matching (PSM) was used to control for variations in baseline characteristics, such as age, tumor stage, lymph node stage, histological grade, and Ki-67%. Subsequently, 1:1 matched data were analyzed to investigate the prognostic differences in DFS between the two groups, which may result in the exclusion of some patients from the cohort. Moreover, the analysis of data and its graphical representation were performed in R Studio (version 4.1.3). For all analyses, a P-value below 0.05 was considered statistically significant.

## Results

### Clinical and pathological characteristics of HER-2-positive BC patients with trastuzumab treatment

The flowchart of the study design can be found in Supplementary Fig. 1. Table 1 summarizes the characteristics of the 544 patients receiving 1 year of trastuzumab treatment. These characteristics included age, tumor size, histological grade, lymph node stage, HR status, and Ki-67%. In total, the median follow-up time was 49.5 months (6–95 months). From the initial data, there were more patients aged 40 or below in the low-SII group relative to the high-SII group (24.3% vs. 16.9%; P < 0.05). However, tumor sizes, lymph node staging, histological grade, HR status, and Ki-67% were similar in all groups (all P > 0.05) despite considerable variations in standardized mean differences (SMD). Additionally, the use of neoadjuvant treatment, adjuvant chemotherapy, endocrine therapy, and radiation therapy showed no differences between the two groups (all P > 0.05) (Supplementary Table 1). As shown in Supplementary Table 1, around 80% of patients received 4 cycles of epirubicin and cyclophosphamide, followed by 4 cycles of docetaxel. Following PSM, differences in age between the groups (P > 0.05) and the SMD values for all factors were effectively balanced (all SMD < 0.10).

**Table 1:** Characteristics of HER-2-positive patients receiving trastuzumab treatment before and after PSM.

Factors	Raw data, N (%)		P-value	SMD	After PSM, N (%)		P-value	SMD
	Low SII	High SII			Low SII	High SII		
Age (year)								
≤ 40	66 (24.3)	46 (16.9)	0.034	0.183	26 (14.8)	26 (14.8)	1.000	< 0.001
> 40	206 (75.7)	226 (83.1)			150 (85.2)	150 (85.2)		
T stage								
T1	52 (19.1)	50 (18.4)	0.637	0.081	26 (14.8)	30 (17.0)	0.840	0.063
T2	182 (66.9)	176 (64.7)			130 (73.8)	126 (71.6)		
T3–T4	38 (14.0)	46 (16.9)			20 (11.4)	20 (11.4)		
N stage								
pN0	154 (56.6)	132 (48.5)	0.125	0.175	90 (51.1)	90 (51.1)	0.828	0.066
pN1	68 (25.0)	74 (27.2)			40 (22.8)	36 (20.5)		
pN2–3	50 (18.4)	66 (24.3)			46 (26.1)	50 (28.4)		
Histological grade								
1–2	230 (84.6)	224 (82.4)	0.489	0.059	152 (86.4)	152 (86.4)	1.000	< 0.001
3	42 (15.4)	48 (17.6)			24 (13.6)	24 (13.6)		
HR								
Negative	144 (52.9)	162 (59.6)	0.120	0.134	108 (61.4)	108 (61.4)	1.000	< 0.001
Positive	128 (47.1)	110 (40.4)			68 (38.6)	68 (38.6)		
Ki-67/%								
< 30%	142 (52.2)	124 (45.6)	0.123	0.133	88 (50.0)	92 (52.3)	0.670	0.045
≥ 30%	130 (47.8)	148 (54.4)			88 (50.0)	84 (47.7)		

*T* tumor size, *N* lymphaden, *HR* hormone receptor, *SMD* standardized mean difference, *PSM* propensity score matching, *SII* systemic immune-inflammation index.

### Survival analysis of patients who underwent trastuzumab treatment

**Figure 1:**
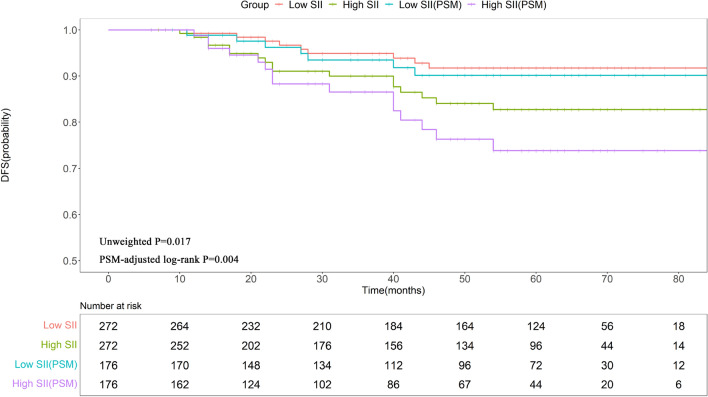
KM curves for DFS in patients receiving trastuzumab. *SII* systemic immune-inflammation index, *PSM* propensity score matching, *DFS* disease-free survival.

During the follow-up, 54 out of 544 patients experienced disease recurrence or metastasis. Prior to PSM, the KM analysis showed that survival differed significantly between the two groups. As presented in Fig. 1, the 3-year and 5-year DFS rates were markedly reduced in high-SII patients relative to those with low SII (89.97% vs. 94.91%; 82.76% vs. 91.76%; P = 0.017) (Fig. 1). Following PSM, the prognosis of the low-SII group was significantly better than that of high-SII participants (P = 0.004) (Fig. 1). As such, a Cox regression model was similarly employed to ascertain factors influencing DFS rates in patients administered with trastuzumab treatment post-PSM. Lymph node involvement (pN1 vs. pN0, HR = 4.250, 95% CI = 1.550–11.680, P = 0.005; pN2-3 vs. pN0, HR = 12.900, 95% CI = 5.390–30.890, P < 0.001) and high SII (HR = 3.430, 95% CI = 1.830–6.420, P < 0.001) were found to be independent risk factors for reduced DFS (Table 2). In contrast, HR positivity independently enhanced the DFS length (HR = 0.150, 95% CI = 0.060–0.360, P < 0.001) (Table 2).

**Table 2:** Univariate and multivariate analyses of DFS before and after PSM in patients treated with trastuzumab.

Factors	Univariate			Multivariate		
	HR*	95% CI	P-value	HR*	95% CI	P-value
Age (year) (> 40 vs. ≤ 40)	0.650	0.320–1.310	0.230			
T stage (2 vs. 1)	1.870	0.650–5.320	0.243	0.820	0.270–2.510	0.724
T stage (3–4 vs. 1)	7.030	2.230–22.110	0.001	1.430	0.410–4.960	0.578
N stage (pN1 vs. pN0)	3.420	1.350–8.700	0.010	4.250	1.550–11.680	0.005
N stage (pN2-3 vs. pN0)	8.760	3.980–19.270	< 0.001	12.900	5.390–30.890	< 0.001
Histological grade (3 vs. 1–2)	1.640	0.760–3.540	0.204			
HR (positive vs. negative)	0.190	0.080–0.450	< 0.001	0.150	0.060–0.360	< 0.001
Ki-67/% (≥ 30 vs. < 30)	1.090	0.610–1.940	0.779			
SII (high vs. low)	2.380	1.300–4.370	0.005	3.430	1.830–6.420	< 0.001

*T* tumor size, *N* lymphaden, *HR* hormone receptor, *PSM* propensity score matching, *SII* systemic immune-inflammation index, HR* hazard ratio, *95% CI* 95% confidence interval.

### Clinical and pathological characteristics of HER-2-positive BC patients without trastuzumab treatment

The raw data indicated that 253 HER-2-positive BC patients did not receive trastuzumab treatment. The median follow-up time was 48.5 months (6–95 months). The high- and low-SII groups were similar in age, tumor size, lymph node staging, histological grade, HR status, and Ki-67% (all P > 0.05) despite substantial differences in SMD (Table 3). Choices for neoadjuvant treatment, adjuvant chemotherapy, endocrine therapy, and radiation therapy also showed no significant differences between the two groups (all P > 0.05) (Supplementary Table 1). The primary chemotherapy regimen involved 4 cycles of epirubicin and cyclophosphamide, followed by 4 cycles of docetaxel. Following PSM, the SMD values for all factors were effectively balanced (all SMD < 0.10), as indicated in Table 3.

**Table 3:** Characteristics of HER-2-positive patients not treated with trastuzumab before and after PSM.

Factors	Raw data, N (%)		P-value	SMD	After PSM, N(%)		P-value	SMD
	Low SII	High SII			Low SII	High SII		
Age (year)								
≤ 40	16 (12.7)	15 (11.8)	0.830	0.027	6 (7.5)	6 (7.5)	1.000	< 0.001
> 40	110 (87.3)	112 (88.2)			74 (92.5)	74 (92.5)		
T stage								
T1	28 (22.2)	22 (17.3)	0.395	0.172	16 (20.0)	16 (20.0)	0.975	0.035
T2	80 (63.5)	80 (63.0)			51 (63.8)	52 (65.0)		
T3–T4	18 (14.3)	25 (19.7)			13 (16.2)	12 (15.0)		
N stage								
pN0	66 (52.4)	77 (60.6)	0.416	0.167	45 (56.2)	45 (56.2)	1.000	< 0.001
pN1	41 (32.5)	34 (26.8)			26 (32.6)	26 (32.6)		
pN2–3	19 (15.1)	16 (12.6)			9 (11.2)	9 (11.2)		
Histological grade								
1–2	98 (77.8)	98 (77.2)	0.907	0.015	66 (82.5)	66 (82.5)	1.000	< 0.001
3	28 (22.2)	29 (22.8)			14 (17.5)	14 (17.5)		
HR								
Negative	71 (56.3)	73 (57.5)	0.856	0.023	48 (60.0)	49 (61.3)	0.871	0.026
Positive	55 (43.7)	54 (42.5)			32 (40.0)	31 (38.7)		
Ki-67/%								
< 30%	63 (50.0)	53 (41.7)	0.187	0.166	35 (43.8)	34 (42.5)	0.873	0.025
≥ 30%	63 (50.0)	74 (58.3)			45 (56.2)	46 (57.5)		

*T* tumor size, *N* lymphaden, *HR* hormone receptor, *SMD* standardized mean difference, *PSM* propensity score matching, *SII* systemic immune-inflammation index.

### Survival analysis of patients not receiving trastuzumab treatment

**Figure 2:**
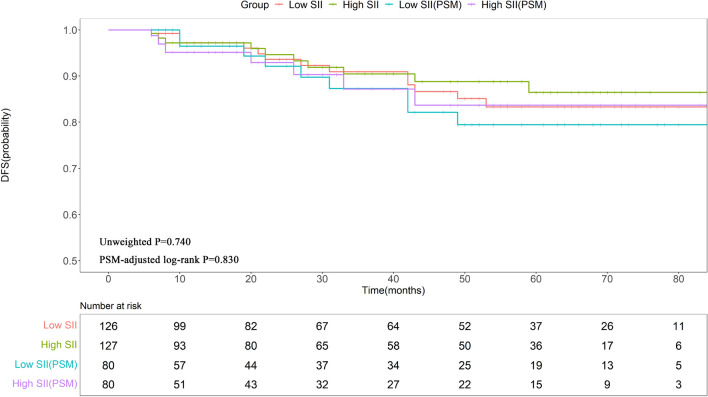
The KM curves demonstrate the DFS outcomes in patients who did not receive trastuzumab treatment. *SII* systemic immune-inflammation index, *PSM* propensity score matching, *DFS* disease-free survival.

During follow-up, 26 out of 253 patients who did not receive trastuzumab treatment experienced metastatic events. From the raw data, the KM curves and log-rank tests in Fig. 2 demonstrated that the 3-year and 5-year DFS were similar between the high- and low-SII groups (90.44% vs. 90.92%; 86.46% vs. 83.32%; P = 0.740). Following PSM, there were still no significant differences in 3-year (87.18% vs. 88.87%) and 5-year (84.05% vs. 83.69%) DFS rates between the groups (P = 0.830) (Fig. 2). Furthermore, multivariate Cox regression analysis of the matched data revealed that only lymph node involvement was independently predictive of DFS (pN 2–3 vs. pN 0, HR = 4.570 95% CI = 1.260–16.590, P = 0.021) (Supplementary Table 2).

### Characteristics of all HER-2-positive BC patients

The total cohort of 797 patients was assigned to four groups, namely, those who did not receive trastuzumab treatment with low SII (Group 1) and high SII (Group 2), participants with low SII values who received trastuzumab (Group 3), and those with high SII scores who trastuzumab treatment (Group 4). Table 4 summarizes the distribution of clinical characteristics over the entire cohort. The median follow-up was 49 months (6–95 months). From Group 3, there were more women aged 40 years or younger (24.3% vs. 12.7% vs.11.8% vs. 16.9%) compared to Groups 1, 2, and 4 (P < 0.05) (Table 4). Furthermore, no differences were observed in terms of tumor size, lymph node staging, histological grade, HR status and Ki-67% (P > 0.05), as presented in Table 4.

**Table 4:** Characteristics of the entire patient cohort.

Factors	Without trastuzumab		With trastuzumab		P-value	SMD
	Low SII	High SII	Low SII	High SII		
Age (year)						
≤ 40	16 (12.7)	15 (11.8)	66 (24.3)	46 (16.9)	0.004	0.184
> 40	110 (87.3)	112 (88.2)	206 (75.7)	226 (83.1)		
T stage						
T1	28 (22.2)	22 (17.3)	52 (19.1)	50 (18.4)	0.762	0.112
T2	80 (63.5)	80 (63.0)	182 (66.9)	176 (64.7)		
T3–T4	18 (14.3)	25 (19.7)	38 (14.0)	46 (16.9)		
N stage						
pN0	66 (52.4)	77 (60.6)	154 (56.6)	132 (48.5)	0.050	0.206
pN1	41 (32.5)	34 (26.8)	68 (25.0)	74 (27.2)		
pN2–3	19 (15.1)	16 (12.6)	50 (18.4)	66 (24.3)		
Histological grade						
1–2	98 (77.8)	98 (77.2)	230 (84.6)	224 (82.4)	0.206	0.114
3	28 (22.2)	29 (22.8)	42 (15.4)	48 (17.6)		
HR						
Negative	71 (56.3)	73 (57.5)	144 (52.9)	162 (59.6)	0.478	0.071
Positive	55 (43.7)	54 (42.5)	128 (47.1)	110 (40.4)		
Ki-67/%						
< 30%	63 (50.0)	53 (41.7)	142 (52.2)	124 (45.6)	0.190	0.120
≥ 30%	63 (50.0)	74 (58.3)	130 (47.8)	148 (54.4)		

T, tumor size; N, lymphaden; HR, hormone receptor; SMD, standardized mean difference; PSM, propensity score matching; SII, systemic immune-inflammation index.

### Survival analysis of the entire cohort

**Figure 3:**
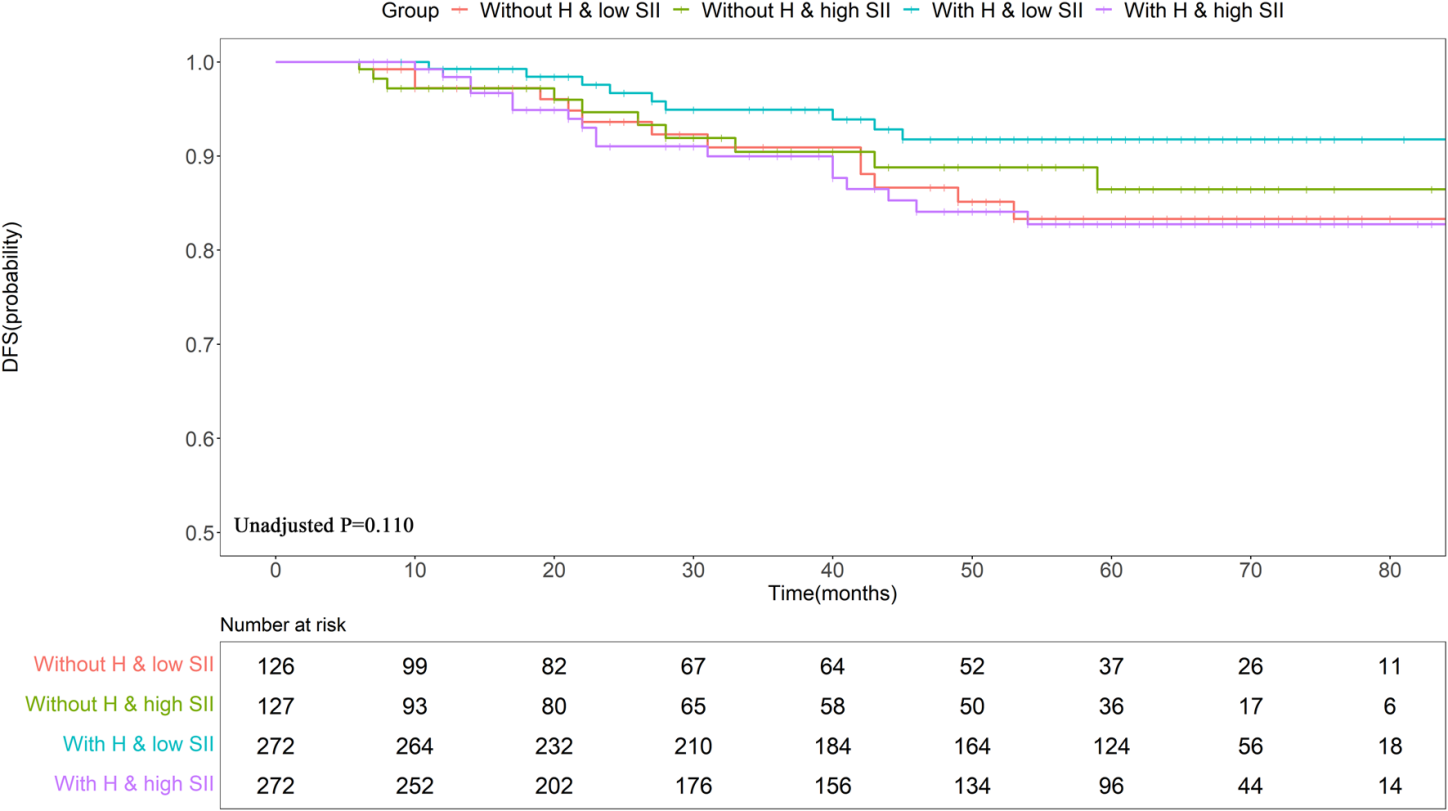
KM curves demonstrating the DFS outcomes in the overall population. *SII* systemic immune-inflammation index, *PSM* propensity score matching, *DFS* disease-free survival, *H* trastuzumab.

KM survival analysis with log-rank tests (Fig. 3) indicated a higher trending 5-year DFS rate in Group 3 relative to that in Groups 1, 2 and 4 (91.76% vs. 83.32% vs. 86.46% vs. 82.76%, P = 0.110). However, the 5-year DFS outcomes for patients in Groups 1, 2, and 4 were similar.

## Discussion

To evaluate the influence of trastuzumab on DFS in HER-2-positive patients, we examined several factors traditionally used in prognosis prediction, such as age, tumor size and grade, lymph node involvement, HR status, and the SII score as a predictor of inflammation. In patients who received trastuzumab, a high SII was found to be independently predictive of poor DFS. However, SII was not found to be predictive in HER-2-positive patients who did not receive trastuzumab. Additionally, the DFS of patients receiving trastuzumab treatment with high SII (Group 4) was similar or slightly inferior compared to those who were not treated with trastuzumab (Groups 1 and 2). Therefore, more information is needed to verify whether SII can predict the population that would benefit from trastuzumab treatment.

SII is a commonly used marker for systemic inflammatory response and is easily accessible. However, the current research value of the SII still primarily stems from retrospective studies. Multiple studies have found that in HER-2-positive BC, high SII is an independent risk factor for DFS, OS, and DMFS^14–18^. Additionally, SII can predict long-term prognosis in breast cancer patients undergoing surgery^15,16^. Moreover, high SII is also an adverse factor for the achievement of complete pathological response in HER-2-positive patients after neoadjuvant chemotherapy^17^. Furthermore, high SII levels are a risk factor for axillary lymph node metastasis in HER-2-positive BC^18^. To date, little is known about the usefulness of SII in predicting the efficacy of trastuzumab treatment.

In this study, we first categorized the participants based on whether they received trastuzumab treatment. Data from patients who were not treated with trastuzumab confirmed that SII had no predictive value. However, data from patients receiving trastuzumab treatment showed an association between low SII values and improved survival rates. However, the specific reasons for this difference are still unclear. Cancer-related inflammation is considered a key mechanism in tumor progression^8^, and tumor-induced inflammatory responses can cause corresponding changes in the blood’s neutrophil, lymphocyte, and platelet counts^19^. Neutrophils are the most abundant white blood cells in human blood. They are also believed to be the first recruited to inflammatory sites and essential components of tumor-associated inflammatory cell infiltration, playing a major part in both the development and progression of the tumor^20,21^. The involvement of platelets in the tumorigenesis of various malignancies has also been demonstrated^22^. Platelets tightly control the actions of neutrophils by forming platelet-neutrophil complexes in the blood and guiding neutrophils into ischemic tissues^23^. Additionally, leaky tumor vasculature allows direct interaction between platelets and tumors. As such, platelets are stimulated by tumor cells, resulting in platelet accumulation and the secretion of factors promoting both the growth and angiogenesis of the tumor^24,25^. Furthermore, lymphocytes are essential components of the tumor immune microenvironment, where they recognize and destroy tumor cells^26^. Notably, tumor-infiltrating lymphocytes (TILs) at the primary tumor site have been linked to improved prognosis^27^. Thus, SII is a systemic marker of both immune function and inflammation that can reflect, to a certain extent, the comprehensive immune status of the body. As mentioned above, the SII is calculated as follows: SII = (neutrophils count × platelets count)/lymphocyte count. A decrease in the SII value means a reduction of the neutrophil and platelet counts and an increase in lymphocyte count, likely indicating the favorable condition of the immune system.

In this study, we found that a low SII value was associated with better DFS in patients receiving trastuzumab therapy but not in those without trastuzumab treatment. Considering that SII is a systemic marker of host immune function, the association of SII and DFS might be explained by the trastuzumab-induced ADCC, which exerts anti-tumor functions and reduces metastasis. Trastuzumab might also induce the ADCC function against metastasis of BC more efficiently if the host immune status is in favorable condition (low SII status). Moreover, trastuzumab might not induce ADCC function if the host immune system is in a suppressive condition (high SII status). In summary, we hypothesize that trastuzumab may exert more substantial anti-tumor immune capabilities through ADCC in patients with low SII relative to those with high SII scores. However, further evidence and research are needed to explore the anti-tumor efficacy of trastuzumab in different immune states.

This study does have certain limitations. First, the study was a single-center retrospective investigation. The data distribution among Groups was not uniform. However, further grouping and PSM were used to balance the impact of confounding factors such as age and lymph node status on DFS outcomes. Overall, it remains a small sample study, and further verification with larger samples is required to support the research conclusions. Secondly, the timing of trastuzumab treatment was not the same in the patient cohort. Some patients received trastuzumab treatment during neoadjuvant chemotherapy, while others received it during adjuvant chemotherapy. Further, the chemotherapy regimens varied among different groups, which might influence the host immune system. Thus, a large prospective study will be needed to solve these problems. Thirdly, we did not analyze other immune-related markers that may influence the efficacy of trastuzumab treatment, such as TILs in the tumor microenvironment. The correlation between SII and TILs deserves further exploration and could provide additional evidence for our research conclusions. Fourthly, to investigate the relationship between SII and trastuzumab-induced ADCC functionality, a reanalysis of classical clinical trials would be necessary. Fifthly, when making multiple group comparisons in the overall population, we did not adjust the P-value for multiple comparisons, which could potentially increase the likelihood of false positives. Lastly, cut-off values for high and low SII groups still vary across different studies, and conducting related prospective studies to determine the optimal cut-off value would have clinical significance.

## Conclusion

This study showed that a lower SII score was predictive of better DFS in patients with HER-2-positive BC undergoing trastuzumab therapy. Importantly, the findings suggest that SII may be helpful in identifying patients likely to benefit from treatment with trastuzumab.

### Supplementary Information

Supplementary Information.

## Data Availability

The data that support the findings of this study are available from the corresponding author, Zhi Xiao, upon reasonable request. The data are not publicly available since this could compromise the privacy of research participants.

## Abbreviations

T: Tumor size
N: Lymphaden
HR: Hormone receptor
SMD: Standardized mean difference
PSM: Propensity score matching
SII: Systemic immune-inflammation index
95% CI: 95% Confidence interval
DFS: Disease-free survival
ADCC: Antibody-dependent cell-mediated cytotoxicity
HR*: Hazard ratio
KM: Kaplan–Meier
TILs: Tumor-infiltrating lymphocytes
