# Supplementary material for: Systemic immune-inflammation index as a prognostic marker in HER2-positive breast cancer patients undergoing trastuzumab therapy
Source: Sci Rep. 2024 Mar 19;14:6578. doi: 10.1038/s41598-024-57343-0 (PMC10951263; doi:10.1038/s41598-024-57343-0)
Supplement: Supplementary file 1 — Supplementary Information. [file 41598_2024_57343_MOESM1_ESM.pdf]

Systemic Immune-Inflammation Index as a Prognostic Marker in HER2-Positive Breast Cancer Patients Undergoing Trastuzumab Therapy

Jian Pang[a], Nianhua Ding[d], Nana Yin[e], and Zhi Xiao[b,c*]

[a] Department of General Surgery, The Second Xiangya Hospital, Central South University, Changsha, China

[b] Department of Breast Surgery, Xiangya Hospital, Central South University, Changsha, China

[c] Clinical Research Center For Breast Cancer In Hunan Province, Changsha, China

[d] Department of Clinical Laboratory, the Affiliated Changsha Hospital of Xiangya School of Medicine , Central South University, Changsha, China

[e] Department of Operating Room, First People's Hospital of Changde, Changde, China

Corresponding Authors

Zhi Xiao, MD, Department of Breast Surgery, Xiangya Hospital, Central South University

Address: 87# Xiangya Road, Changsha City, Hunan Province, P. R. China. 410008. Email: zhixiao@csu.edu.cn

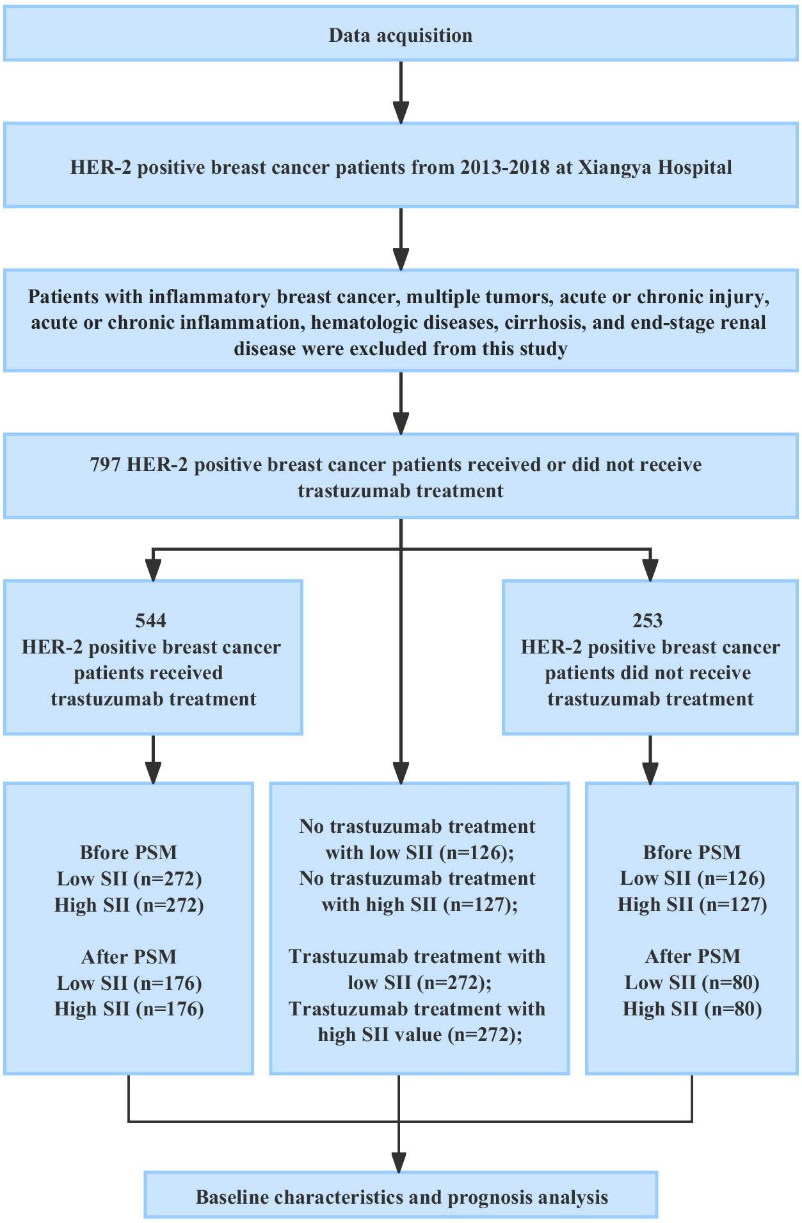

Supplementary Figure 1: The flowchart of the study design.

Supplementary Table 1. Treatment for HER2-positive BC patients.

Factors	Without trastuzumab				With trastuzumab			
	Low SII	High SII	P-value	SMD	Low SII	High SII	P-value	SMD
Neoadjuvant chemotherapy								
No	60(47.6)	56(44.1)	0.574	0.071	120(44.1)	126(46.3)	0.605	0.044
Yes	66(52.4)	71(55.9)			152(55.9)	146(53.7)		
Chemotherapy regimen								
EC-T	99(78.6)	101(79.5)	0.909	0.093	231(84.9)	221(81.2)	0.618	0.115
TCb	18(14.3)	19(15.0)			29(10.7)	35(12.9)		
P*	4(3.2)	4(3.1)			6(2.2)	10(3.7)		
Others	5(4.0)	3(2.4)			6(2.2)	6(2.2)		
Radiotherapy								
No	61(48.4)	52(40.9)	0.232	0.151	119(43.8)	133(48.9)	0.229	0.103
Yes	65(51.6)	75(59.1)			153(56.2)	139(51.1)		
Endotherapy								
No	74(58.7)	66(52.0)	0.279	0.136	154(56.6)	144(52.9)	0.389	0.074
Yes	52(41.3)	61(48.0)			118(43.4)	128(47.1)		

Abbreviations: SMD, standardized mean difference; SII, systemic immune-inflammation index; EC-T, Epirubicin, Cyclophosphamide, and Docetaxel; TCb, Docetaxel and Carboplatin; P*, Paclitaxel.

Supplementary Table 2. Univariate and multivariate analyses of DFS before and after PSM in patients treated without trastuzumab.

Factors	Univariate			Multivariate		
	HR*	95%CI	P-value	HR*	95%CI	P-value
Age (year) (>40 vs. ≤40)	1.210	0.160-9.200	0.855			
T stage (2 vs. 1)	-	-	-			
T stage (3-4 vs. 1)	-	-	-			
N stage (pN1 vs. pN0)	1.600	0.490-5.250	0.436	1.810	0.550-5.960	0.327
N stage (pN2-3 vs. pN0)	6.490	1.810-23.250	0.004	4.570	1.260-16.590	0.021
Histological grade (3 vs. 1-2)	0.400	0.050-3.050	0.378			
HR (positive vs. negative)	0.190	0.040-0.820	0.027	0.220	0.050-1.000	0.050
Ki-67/% (≥ 30 vs. <30)	0.970	0.350-2.690	0.959			
SII (high vs. low)	1.110	0.400-3.080	0.835			

Abbreviations: T, tumor size; N, lymphaden; HR, hormone receptor; PSM, propensity score matching; SII, systemic immune-inflammation index; HR*, hazard ratio; 95% CI, 95% confidence interval.
